# Rhythm in speech and animal vocalizations: a cross‐species perspective

**DOI:** 10.1111/nyas.14166

**Published:** 2019-06-25

**Authors:** Andrea Ravignani, Simone Dalla Bella, Simone Falk, Christopher T. Kello, Florencia Noriega, Sonja A. Kotz

**Affiliations:** ^1^ Artificial Intelligence Laboratory Vrije Universiteit Brussel Brussels Belgium; ^2^ Institute for Advanced Study University of Amsterdam Amsterdam the Netherlands; ^3^ International Laboratory for Brain Music and Sound Research (BRAMS) Montréal Quebec Canada; ^4^ Department of Psychology University of Montreal Montréal Quebec Canada; ^5^ Department of Cognitive Psychology Warsaw Poland; ^6^ Laboratoire de Phonétique et Phonologie, UMR 7018, CNRS/Université Sorbonne Nouvelle Paris‐3 Institut de Linguistique et Phonétique générales et appliquées Paris France; ^7^ Cognitive and Information Sciences University of California Merced California; ^8^ Chair for Network Dynamics Center for Advancing Electronics Dresden (CFAED), TU Dresden Dresden Germany; ^9^ CODE University of Applied Sciences Berlin Germany; ^10^ Basic and Applied NeuroDynamics Laboratory, Faculty of Psychology and Neuroscience, Department of Neuropsychology and Psychopharmacology Maastricht University Maastricht the Netherlands; ^11^ Department of Neuropsychology Max‐Planck Institute for Human Cognitive and Brain Sciences Leipzig Germany

**Keywords:** speech rhythm, hierarchical, timing, time perception, rhythm cognition, bioacoustics

## Abstract

Why does human speech have rhythm? As we cannot travel back in time to witness how speech developed its rhythmic properties and why humans have the cognitive skills to process them, we rely on alternative methods to find out. One powerful tool is the comparative approach: studying the presence or absence of cognitive/behavioral traits in other species to determine which traits are shared between species and which are recent human inventions. Vocalizations of many species exhibit temporal structure, but little is known about how these rhythmic structures evolved, are perceived and produced, their biological and developmental bases, and communicative functions. We review the literature on rhythm in speech and animal vocalizations as a first step toward understanding similarities and differences across species. We extend this review to quantitative techniques that are useful for computing rhythmic structure in acoustic sequences and hence facilitate cross‐species research. We report links between vocal perception and motor coordination and the differentiation of rhythm based on hierarchical temporal structure. While still far from a complete cross‐species perspective of speech rhythm, our review puts some pieces of the puzzle together.

## Graphical abstract

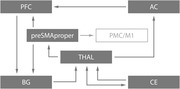

## Introduction

The comparative, cross‐species approach is a powerful method to understand the evolution of cognitive and communicative traits in our species.^1^ Here, we use this approach to study vocal rhythm and investigate which similar traits can be found in other species, so to understand what is broadly shared across, for example, mammals, tetrapods, or vertebrates. We review several studies in the literature that are usually unconnected (see Table 1 for a glossary). In particular, we discuss the production and perception of rhythmic patterns in nonhuman species and in human development. We summarize several methods to measure rhythmic structure in vocalizations produced by humans and other species. We discuss the neural bases of speech rhythm, attempting to draw comparative links.

**Table 1:** Alphabetical glossary of key terminology^a^

Term	Definition
Auditory grouping	While sounds are perceived as coming from a single source, similar sounds tend to group together
Babbling	Vocal experimentation and sound practice found in the early developmental stage of select species. In human infants, babbling precedes the emergence of first words (starting around 4−5 months of age, with variable duration, and lasting until the second and even third year of life)
Beat	Psychological process resulting in the perception and extraction of an isochronous pulse (not necessarily present in the physical signal) from a rhythmic sequence. The perception of a beat can result from metrical expectations, associated with different embedded periodicities
Durational categories	Classification or perception of different temporal intervals not as a continuum but as each belonging to a particular category. An indirect way of detecting durational categories in a dataset of durations is testing whether the distribution of durations is not uniform but multimodal, where each mode approximates the prototype of one category
Frontostriatal brain circuitry	Neural pathway(s) connecting cortical frontal lobe brain areas with the striatum, including the putamen and caudate nucleus
Grouping	Process of building basic patterns of sound events based on acoustic features, such as stress, loudness alternation, pitch variation, durational relationships, etc.
Hierarchical temporal structure	Clustering of signal events in time, such as peaks in the amplitude envelope, where smaller clusters are nested within larger clusters across timescales
Iamb	“Metrical form in speech alternating a weak (unstressed) syllable with a strong (stressed) syllable”
(Inter‐event) interval	“Temporal duration encompassed by two events.” Examples of events are the onset and offset times of a vocalization
Isochronous	“A series of events repeating at a constant rate”
Meter	Hierarchical organization of patterns of events based on spectral and structural properties
Rhythm	“Pattern of events in time,” possibly with “a specific succession of durations” and accents as seen in speech
Stuttering	A developmental speech fluency disorder, which interrupts the rhythmic flow of speech and communication^9^
Timing	Perception and production of temporal relationships
Trochee	“Metrical form in speech alternating a strong syllable with a weak syllable”

a Definitions in quotation marks are reproduced verbatim from Refs. 7 and 8.

Rhythm processing requires (but is not limited to) the ability to produce and perceive individual temporal intervals. Hence, we set off by briefly discussing literature on interval timing. Thorough treatments of interval timing are available elsewhere (see Refs. 2−6).

## Human and nonhuman studies of vocal rhythm

### Animal timing from the psychophysics literature

Timing and time perception has a long tradition in animal research. Rats, mice, pigeons, fish, and some primate species have all been studied in terms of their ability to estimate or reproduce temporal intervals in the millisecond‐to‐second range. A general finding of these studies is that predictions from the so‐called scalar expectancy theory hold across species and domains (with some exceptions, see Ref. 4). Simply put, the theory predicts that timing sensitivity, corresponding to the accuracy in perceiving or reproducing time intervals, is inversely proportional to interval duration: animals, including humans, estimate longer intervals with less accuracy.

Research on timing and time perception is necessary but not sufficient to understand rhythm (for a parallel in music, see Ref. 10). In fact, timing research often focuses on the production or perception of individual time intervals. Rhythm instead focuses on patterns of temporal events, whose building blocks are individual time intervals. This is similar to perceiving individual frequencies that can be understood as a building block for perceiving the harmonies and timbres of sounds. However, the perception of individual frequencies is not sufficient to understand the perception of multiple overlapping frequencies. For instance, if humans listen to two tones at particular frequencies, they will hear a third one (the “missing fundamental”^11^) whose presence cannot be predicted by basic psychophysical data on individual frequency. Similarly, as reviewed here, simple timing patterns can be layered to create the perception of rhythmic structure that is not simply determined by its components (e.g., see Ref. 12).

### Comparative experiments: training and testing animals on rhythm, meter, and prosody

Rhythm involves a series of time intervals, often at multiple timescales, that can combine to produce a hierarchical metrical structure.^13^ The perception of rhythmic features, such as grouping, is usually studied in operant experiments.^14^ Rats, budgerigars, and zebra finches have recently been tested in their capacity for metrical grouping. Rats, like humans, are capable of using pitch alternation in sound sequences to group them as trochees (high−low pairs); in contrast, unlike humans, rats cannot use durational alternation in sound sequences to group them as iambs (short−long pairs).^15^ Zebra finches show similar discrimination capacities as rats.^16^ Follow‐up work has shown that, if thoroughly trained for a durational alternation, rats can indeed discriminate between iambs and trochees.^17^ In a related experiment, although within a different setup, budgerigars could distinguish between iambic and trochaic meter, but required, to succeed, more than one cue among pitch, duration, loudness, and vowel quality.^18^ Testing rats with stimuli identical to those used for budgerigars revealed a very different result: unlike parrots, rodents need all four cues to discriminate between prosodic patterns. Of these four cues, one was purely (duration) and two partly (loudness and pitch) rhythmic.^17^ In summary, the ability to perceive and discriminate a simple metrical structure has been observed in several species, but more research is needed to fully determine the extent of these abilities across species.

### Spontaneous individual vocal rhythms: what kind of temporal structure is contained in animals’ call sequences and songs?

Several species have also been found to produce spontaneous vocal rhythms and are therefore particularly promising for comparative human–animal research,^19^ including (1) laboratory rodents, such as mice, because biomedical research has thoroughly mapped their neurobiology;^20^ (2) nonhuman primates, because of their phylogenetic relatedness to humans;^21^ (3) songbirds, in particular, zebra finches, because they are an established model species for avian vocal flexibility and learning;^22^ and (4) vocal learning mammals, such as seals, elephants, and bats, because they are the closest vocal learning animals to humans.^23^ Below, we will briefly discuss examples of vocal rhythms in rodents, nonhuman primates, songbirds, and mammalian vocal learners.

**Figure 1:**
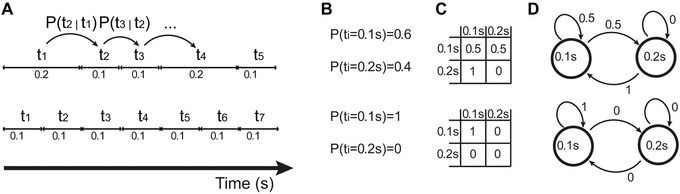
Some of the possible ways of representing temporal patterns (bottom: isochronous, top: nonisochronous). (A) Time series of intervals, inducing transition probabilities, such as P(t_2_|t_1_), which means the probability that the (inter‐event) interval t_2_ of length *x* ms follows an interval t_1_ of length *y* milliseconds. (B) Individual probabilities of occurrence of a particular durational interval. (C) Transition matrices based on the transition probabilities described before. (D) A probabilistic finite state machine, which can also generate durational patterns as those seen in A and summarized in the transition matrices in C. Figure reproduced verbatim from Ref. 8, an Open Access article distributed under the terms of the Creative Commons Attribution License (CC BY).

Measures of vocal rhythm can be found in “transition probabilities”; these probabilities have become popular in birdsong and language research. Given a sequence of events, including event types A, B, C, etc., the transition probability between event types A and B is the probability that A is followed by B. A common application of this concept is to study sequences of discrete elements: in birdsong research, a low transition probability between notes A and B means that note A is rarely followed by B. With respect to measuring vocal rhythms, transition probabilities can be used in the temporal domain (see Ref. 8), with the caveat that time is continuous, so some discretization is necessary. For instance, one could calculate the transition probability from short to long call durations and vice versa (Fig. 1); if the former was high and the latter was low, short calls would often be followed by long calls, but long calls rarely would be followed by short calls.

In mice, ultrasonic vocalizations exhibit quite stable transition probabilities in durations, especially for short‐short and long‐long transitions.^20^ This temporal structure could be summarized by matrices as those in Figure 1C with values close to 1 in the diagonal, and values close to 0 elsewhere. Mice vocalizations also appear to be temporally organized in a hierarchical fashion.^20^ However, more (operant) work is needed to test the existence of hierarchical organization at a neurocognitive level, that is, temporal events structured at different time scales, possibly embedding one level into the higher one, as opposed to structure appearing hierarchical as a byproduct of serial behavior or anatomical constraints (see Refs. 24 and 25 for a parallel discussion about recursion and cognition in behavior). Finally, taking a developmental perspective, the rhythm of mice vocalizations as pups is predictive of vocal rhythms in the same mice as adults.^20^ These results suggest that there may be sensitive phases for rhythm development in infant mice, assuming that some learning is involved in mice vocal rhythms. Unlike common mice, Alston's singing mice do perform vocal duets: in this neotropical rodent, call timing is controlled by different neural circuitry depending on whether singing is performed in isolation or socially.^26^, ^27^, ^28^

Research on individual vocal rhythms in nonhuman primates is scarce: most work has investigated either group vocal rhythms^29^, ^30^ or individual nonvocal rhythms.^31^, ^32^, ^33^ Focusing on individual vocal rhythms, early descriptive work remarked temporal regularities in gelada monkeys’ vocalizations,^34^ a claim which is intriguing but purely descriptive, unfortunately not supported by quantitative data or statistical inference. More recent work in macaques and orangutans noted a 5‐Hz isochronous pattern during lip‐smacking, facial movement, or vocalization.^35^, ^36^, ^37^ Primate perspectives on speech rhythm can be found elsewhere (e.g., see Ref. 21), but it is clear that we do need to understand more about vocal rhythms in our closest living relatives, the primates.

**Figure 2:**
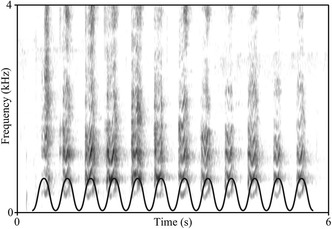
Spectrogram showing the isochronous barking of a California sea lion. One possible way to visually detect isochronous regularity is to superimpose a metronomic grid, like the regular sinusoidal function shown here, to the spectrogram. Figure reproduced verbatim from Ref. 8, an Open Access article distributed under the terms of the Creative Commons Attribution License (CC BY).

Zebra finches have long been a model for vocal learning, though research in this species has historically focused on the spectral and combinatorial domains, rather than the temporal and rhythmic domains. The temporal dimension of zebra finches’ songs has been explored recently, and the rhythms of their songs appear to be characterized by plasticity and interindividual variability, which are connected to learning and often in contrast to stereotypical calling.^38^ Past methods used in birdsong research concluded strong stereotypy in zebra finches’ rhythms, but this conclusion may have stemmed from analytical methods that focus on short time scales to the neglect of longer time scales.^38^ We now find that zebra finches can flexibly time their unlearned calls.^39^ In addition, zebra finches’ songs exhibit a form of isochronous regularity: syllable onsets coincide, more often than not, with regular “beats” of an idealized isochronous grid (Fig. 2).^22^ Evidence for the interplay between plasticity and regularity makes intuitive sense: an underlying isochronous grid can provide anchor points in time from which songs can be learned, structured, and flexibly varied.

The isochrony detection technique used in zebra finches has also been applied to a bat species capable of vocal production learning. Surprisingly, the neotropical bat *Saccopteryx bilineata* exhibits isochronous rhythms not only in its echolocation calls, but also in male vocal displays (i.e., “songs”) and pups’ call sequences.^40^ In addition, a (post‐hoc) superimposed metronomic grid exhibited a tempo, which matched the wing‐beat of the animals.^40^ The finding of temporal similarities between vocal (calls) and nonvocal (wing‐beat) rhythms in bats is one more comparative piece of evidence of the cross‐modality of rhythm, which in humans entails audition, vision, movement, etc. (e.g., dance).

**Figure 3:**
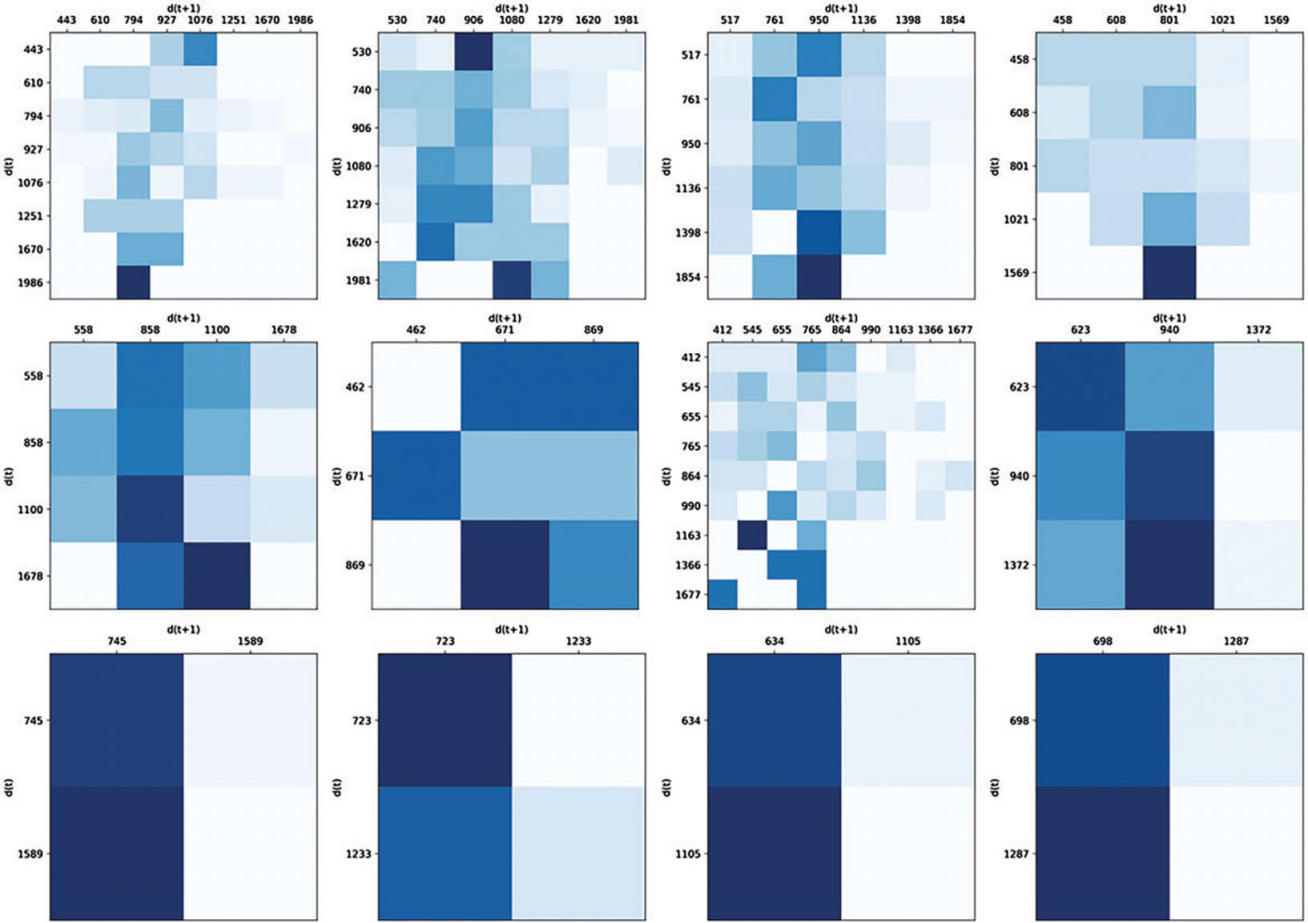
Transition matrices for one individual seal pup across days, each showing the probability that a call of a given duration is followed by another call of the same (diagonal) or another duration. Each matrix represents 1 day, with calendar days progressing from left to right and top to bottom. Each row and column of one matrix represents the centroid of a durational category in milliseconds: leftmost columns and upmost rows are shorter (400−700 ms) categories; the further down and right, the longer the category. Shades of blue represent transition probabilities; that is, the probability, within a sequence of seal pup calls, that a specific category on the vertical axis is followed by a specific category on the horizontal axis. Darker blue corresponds to a higher transition probability; for instance, in the bottom‐right matrix, the dark square means that an element of a durational category centered at 1287 ms is very likely to be followed by an element of a durational category centered at 698 ms, but very unlikely to be followed by an element of the same category centered at 1287 milliseconds. Notice how, over days, the number of categories shrinks (though see Ref. 44) and the transitions from one to the other become more predictable. Figure cropped from Ref. 44, an Open Access article distributed under the terms of the Creative Commons Attribution Non‐Commercial License; additional details in the original paper.

Collecting recordings of some species can be particularly challenging, as in adult male seals that “sing” underwater.^41^ Underwater recordings consist of few microphones recording multiple, nonvisible sources, making it often difficult to attribute vocalizations to individuals.^41^, ^42^ In these cases, one can still indirectly test for rhythmicity by probing whether temporal structures of different song elements covary.^43^ Alternatively, seal pups are often easier to record, as they mostly vocalize on land (as opposed to underwater). Analyses of temporal features of seal pups’ vocalizations have shown some regularities and the emergence of durational categories over development, but larger sample sizes are needed to generalize (Fig. 3; see Ref. 44).

### Babbling and stuttering

Vocal learners, such as harbor seals and *S. bilineata* bats,^45^, ^46^ are useful model species to study the potential interplay between rhythm and vocal learning ontogeny.^38^, ^44^ They may also give directions for research on early human vocal production. For instance, some species share similarities with humans in their earliest vocalizations called “babbling.” Across different language contexts, human infants in their first year of life vocalize rhythmic chunks of repeated and then varied syllables (like da‐da‐da; e.g., see Refs. 47 and 48). Babbling features native language sound production and imitation of prosodic aspects of adult speech (e.g., see Refs. 48 and 49). Babbling as a kind of vocal play and imitation of adult calls, barks, trills, and songs is also observed in infant and juvenile pygmy marmosets,^50^ sac‐winged bat pups,^40^, ^51^ giant otters,^52^ and zebra finches.^53^ According to the Frame and Content theory,^54^ babbling in human infants is a rhythmic motor training, which lays the grounds for basic syllable structure. Infants learn that vocalizing at different times during quasiperiodic cycles of mandibular opening and closure results in vowels, at maximal mandibular opening, and consonants, at maximal mandibular closure. However, it is still unclear how these early syllable rhythms in babbling contribute to later adult rhythms or later language capacities in general.^55^ Results from nonhuman animals suggest that animal babbling is not linked in a simple way to later adult vocal production. For example, female sac‐winged baby bats, during the babbling period, produce adult male songs and trills without producing them as adults.^51^ In zebra finches, different brain circuits are active during juvenile babbling and later adult song production.^53^ These results may inspire future research into human infant babbling by investigating the potential significance of early imitation capacity for later speech perception or the potential differences between neuronal circuits that are active during babbling and early/later speech production.

Finally, potential parallels between humans and nonhuman animals can be investigated in rhythm disorders in early vocal production. Stuttering, for example, is a speech fluency disorder typically emerging between the second and fourth year of life in humans.^56^ Children show untypical disfluencies during speech production, such as silent blocks, syllable and sound repetitions, and prolongations. Stuttering‐like behavior can also be observed in songbirds, such as zebra finches.^57^, ^58^ In humans, recent research links the disturbance of the rhythmic flow of speech in stuttering to faulty auditory−motor learning and erroneous temporal predictions, potentially originating from altered connectivity in subcortical−cortical timing circuits.^59^, ^60^, ^61^ Interestingly, animal research points to a prominent role of basal ganglia dysfunction in stuttering zebra finches,^62^ paralleling findings of impaired basal ganglia functioning in human children and adults who stutter.^63^, ^64^ More research is needed to unravel similarities in how rhythm contributes to the development of skilled speech motor control across species.

### Vocal−motor entrainment in music and speech

Humans are generally highly skilled at processing complex temporal patterns, such as music and speech. Most humans can perceive the regular beat of music, and detect stress in spoken utterances.^65^ Notably, beat perception is often accompanied by a synchronized motor response. For example, the temporal features of musical patterns and their temporal regularity are particularly conducive to movement.^66^ Our proclivity to move to music manifests when we move to its beat, which can happen spontaneously or deliberately, by foot or hand tapping, and in dance or synchronized walking. These skills are widespread in the general population.^67^, ^68^ A compelling body of evidence from experimental psychology and cognitive neuroscience indicates that rhythm and movement are tightly linked.^69^, ^70^, ^71^, ^72^ Matching movements to a beat is possible because the temporal dynamics of rhythmic sound lead to the perception of the beat,^73^ a process linked to internal neurocognitive self‐sustained oscillations.^74^, ^75^ The underlying process, called *entrainment*, generates temporal expectancies, which drive motor control, by allowing the alignment of movements to anticipated event times.

The human ability to spontaneously synchronize to music^76^ and to simpler rhythmic stimuli^77^ contrasts with the lack of evidence on spontaneous motor synchronization in spoken utterances (though see Refs. 78 and 79). Yet, there is some evidence that the accent structure of rhythmical speech, as found in, for example, children's poetry, can entrain movement even when participants are not explicitly instructed to move to speech.^66^ Prominences in speech (stress patterns), akin to musical beats, may indeed represent a target of synchronized movement. Speech rhythm is particularly salient in poems, songs, and children's games (“metrical speech”), characterized by words and phrases that are molded into regularly recurring metrical patterns.^80^, ^81^ For example, in English or German, rhythm is conveyed by accentual patterns whereby strong and weak positions are filled by prominent (i.e., stressed, see Ref. 82) and nonprominent (i.e., unstressed) syllables. Like in music, speech patterns can evoke a subjective impression of isochrony.^83^ This observation is striking, though, given that interstress intervals are typically quite variable in speech (coefficients of variations >30% of the average interstress interval^84^, ^85^), as compared with expressive music (around 10–30% for interbeat intervals in performed expressive music^86^). Moreover, speech meter in conversational speech is clearly less strict and regular than musical meter.^87^ Higher regularity is found in metrical speech, however, such as poetry,^88^, ^89^, ^90^, ^91^ and group speech production, such as prayers and chanting (i.e., choral speaking^92^).

In spite of the higher variability of temporal patterns in speech compared with music, the temporal dynamics of metrical speech can still induce expectations about upcoming events.^93^, ^94^ The substrate of this mechanism lies in the quasi‐rhythmic properties of the speech signal that engage oscillatory behavior in the brain.^95^ Like music, speech patterns are thus capable of driving dynamic attending,^73^ underpinned by neurocognitive self‐sustained oscillations,^93^, ^96^ which phase‐lock to the temporal dynamics of syllabic nuclei in speech.^5^, ^94^, ^97^, ^98^ Accurate prediction of the next verbal event (a stressed syllable) affords a certain degree of motor synchronization to the prominent stress pattern in speech, as observed in recent finger tapping studies.^85^, ^99^, ^100^ Interestingly, concurrent synchronized movement can enhance verbal expectations, as found in prosodically diverse languages, such as German (a lexical stress language) and French (a non‐stress language).^99^, ^101^ For example, finger tapping aligned to accented syllables of spoken utterances benefits the encoding and detection of subtle word changes.^99^, ^101^ Thus, coupling movement to the temporal dynamics of metrical speech can enhance verbal processing and memorization. This effect is reminiscent of more ecological situations in which hand clapping or stamping to metrical speech (e.g., children's lore)^102^ is part of games that may enhance children's social and verbal skills.^103^ Moreover, the aforementioned effects of synchronized movement may pave the way to innovative rhythm‐based interventions currently under investigation for fostering language acquisition and learning in developmental populations with speech and language disorders, such as dyslexic^104^ or autistic children.^105^

The link between rhythm and movement and the ability to couple movement to auditory prominences is ubiquitous in humans. This ability requires little learning, is associated with high flexibility, as humans can adapt to a wide range of tempos even quite far from their preferred movement rates, occurs within a variety of rhythmic stimuli, simple and complex rhythms, and also crossmodally.^106^, ^107^ The question as to whether other species are capable of synchronization to the beat, and if so, to what extent as compared with humans, has fueled research in the last decade. One intriguing hypothesis (the vocal learning—beat perception and synchronization hypothesis^87^, ^108^) postulates that synchronization to a beat is a byproduct of the vocal learning mechanisms that are shared by several bird and mammal species, including humans. In keeping with this hypothesis, a strong link between motor and auditory brain areas is expected to underpin both vocal production and synchronization. There is evidence that these abilities are linked in humans.^109^ This hypothesis received support by the finding that nonhuman animal species, namely sulfur‐crested cockatoos^110^, ^111^ and other bird species that are vocal learners, can also synchronize.^112^, ^113^ Motor synchronization in vocal learners is quite flexible (i.e., adapting to a wider range of tempos), occurs with complex auditory signals, and is crossmodal,^110^, ^111^ thus displaying some of the properties of human synchronization. Recent evidence shows, however, that synchronization to a beat may extend to nonvocal learning species. There is evidence that a chimpanzee can tap above chance, though quite inflexibly, with a 600‐ms metronome,^114^, ^115^ and a California sea lion can bob her head to the beat of a variety of auditory stimuli.^116^, ^117^ Thus, whether synchronization to beat is selectively associated with vocal learning across species is still an open question.^118^, ^119^

### Interactive rhythms during human speech development

As there are some parallels in the development of human and animal rhythmic vocalizations, the question arises to what extent vocal rhythms in interaction are also comparable across species. Only certain animals, though, utter specific pup‐directed vocalizations by making them shorter, more repetitive, or more specialized than adult‐directed vocalizations (see, Ref. 120 for male zebra finches; Ref. 121, for free‐ranging female rhesus macaque; and Ref. 122, for North Atlantic right whale mother–calf pairs). In humans, vocal style changes dramatically in infant–adult interaction. There are at least two functions of rhythmic structure of human infant–adult interaction that may play a pivotal role for infants and young children to acquire speech and language skills: (1) rhythmic vocalizations and imitation subserving *communicative alignment* in early parent–infant interaction, and (2) *temporal predictions* about linguistic structure derived from rhythmic cues in infant‐directed communication. These aspects could be further explored in the animal domain.

Older interlocutors across cultures display a distinct infant‐directed speech register, no matter if they are female, male, parent, sibling, or a stranger. Their utterances are shorter and higher pitched, and they contain distinct melodic contours and more repetition.^123^, ^124^, ^125^ These salient alterations in speech, as well as songs, chants, and rhythmic vocal play^126^, ^127^ contribute to an overall highly rhythmic character of infant‐directed communication. According to evolutionary hypotheses, rhythmic traits of adult–infant interaction are an ancestral part of human child‐rearing practice, whose primary goal was to foster infants’ and mothers’ capacity to affiliate and align with each other and to develop mutual understanding and experience sharing beyond symbolic communication.^128^ In line with this idea, Jaffe and colleagues^129^ found that infant's attachment (at 12 months) is predicted by temporal coordination patterns in turn‐taking with familiar and especially unfamiliar adults at 4 months of age. Overall, from the age of 2 months on, turn‐taking structure between mother and infant vocalizations is already observable with only a 30–40% overlap between reciprocal vocalizations. The most frequent exchange structure features two to three turns, and pauses (gaps) under 1 second.^130^, ^131^

Mutual alignment is considered a key aspect of adult verbal interaction.^132^ Early rhythmic and temporal alignment between mothers and preverbal infants could hence be a precursor of the sophisticated verbal alignment skills needed in later life. In a 2‐year longitudinal study on mother−infant coordination, Abney and colleagues^133^ identified a hierarchical temporal structure as a key aspect of alignment patterns in mother−infant interaction. Hierarchical temporal structure (see below) was extracted from the waxing and waning of amplitude in the acoustic signal thereby identifying hierarchically nested bouts of temporal clusters across timescales. Generally, mothers emphasize the hierarchical temporal structure of infant‐directed speech and singing compared with adult‐directed communication.^134^ Abney and colleagues^133^ found that the hierarchical temporal structure of mothers and infants’ vocalizations was well aligned during mother−infant interactions. In addition, preverbal vocalizations of infants (e.g., vocalic and syllabic sequences) were overall temporally better coordinated with their mother's vocalizations than any nonverbal vocalization (e.g., laughter and cries).

Adult listeners use temporal predictions to better attend to and process phonological, lexical, semantic, and syntactic structure in their interlocutor's speech.^135^, ^136^, ^137^, ^138^ Higher repetitiveness, greater metrical stability, shorter utterances, and enhanced utterance‐final lengthening in infant‐directed speech are all temporal cues, which could help infants to generate *temporal predictions about upcoming linguistic structure*. In infant‐directed speech, temporal cues particularly emphasize phrase boundary information through enhanced preboundary lengthening and longer postboundary pauses.^124^, ^139^ These cues provided by adults help infants to direct their attention to phrase edges. Indeed, infants at 8 months of age more easily segment words at phrase‐final versus medial positions in speech.^140^ Infants are also able to generate temporal predictions from a regular beat structure, such as found in music.^141^, ^142^, ^143^ As a musical stimulus, infant‐directed singing may particularly support beat‐related predictions in caregiver–infant communication. It features clearer metrical structure than speech^126^ and therefore may better direct infants’ attention toward words associated with a beat. First results showed a trend for infants at 11 months of age to process word‐related information in song better than in speech.^144^ Rhythmic structure may also facilitate infants’ discrimination of infant‐directed singing from speech,^145^ and foster the development of more musical and more speech‐related sound processing. Yet, unique contributions of the rhythm of singing to infants’ language and musical skills still await further investigation.

## Techniques for quantifying rhythmic structure

### Rhythm as temporal hierarchy in human and nonhuman vocalization

Rhythm and timing in speech, as in complex animal vocalizations, have hierarchical temporal structure. We know where this structure comes from in speech: units of perception and production are built up hierarchically.^146^ Phonemes are grouped together to form syllables, which are grouped together to form words, which are grouped together to form phrases, and so on. We have many ways of knowing about units of speech perception and production, including behavioral and neural experiments, linguistic inquiry, and our own intuitions. We know much less about the hierarchical structure of animal vocalizations because we do not have the luxury of linguistic inquiry and intuition and experimental methods are limited relative to speech. As a result, we do not have *a priori* units of perception and production that we can map onto recordings of animal vocalizations, as we can with speech recordings, although various methods for segmenting animal vocalizations have been studied.^147^, ^148^, ^149^

Regardless of whether we know the units or not, we can measure and quantify hierarchical temporal structure directly in the acoustic signal that results from vocalization. This structure is different from symbolic hierarchical expressions, as in linguistic research symbolic expressions do not specify timing or temporal durations. Linguistic representations must be elaborated to include temporal structure, which is influenced by the durations of linguistic units, and also prosodic factors such as stress and intonation. Most generally, smaller linguistic units correspond with shorter units of perception or production, which are sequenced together to form larger units, with the possibility of longer durations between larger units. This elaboration only indicates probabilistic, relative relations in temporal structure (Fig. 3), but it leads us to quantitative metrics that we can measure in the acoustic signal.

In particular, we can quantify the *degree* of hierarchical temporal structure. By doing so, we can show an indirect relationship with the putative linguistic units expressed as nested speech units, without needing to map individual units onto specific segments of the speech signal. With this indirect relationship established, we can quantify the degree of hierarchical temporal structure in recordings of animal vocalizations using the same method, and thereby compare the rhythmic structures of speech and animal vocalizations to learn more about their similarities and differences.

Hierarchical temporal structure in the acoustic signals of speech and animal vocalizations can be measured through the amplitude envelope,^150^ which quantifies the bursts and lulls in acoustic energy. The timing and duration of the bursts are captured by clustering in peak events in the amplitude envelope across a wide range of timescales. Smaller clusters are nested within larger clusters across timescales, and nesting can be quantified using Allan factor (AF) variance.^151^ AF variance measures temporal clustering of events at a given timescale by measuring variance in event counts for adjacent time windows. AF functions are computed by measuring AF variance across a range of timescales, to gauge the degree to which clustering increases across timescales.

Falk and Kello^134^ analyzed recordings of German mothers either singing a song or telling a story to their infants, compared with the same mothers singing or storytelling to adults. AF functions showed a greater degree of nested clustering in infant‐directed versus adult‐directed speech and song, particularly in timescales ranging from hundreds of milliseconds to more than 10 seconds. Follow‐up analyses showed that AF functions reflected the greater degree of prosodic exaggeration in infant‐directed speech. Prosodic exaggeration is known to increase the variability in the acoustic durations of units of speech production, and AF variance captures this variability across a range of timescales. The authors analyzed hand‐coded durations of linguistic units ranging from syllables to words and phrases to overall variability in speaking rate. The slopes of AF functions were shown to account for significant variability in all these linguistic units. This result provides supporting evidence that hierarchical temporal structure maps onto linguistic units as they are expressed in speech production.

**Figure 4:**
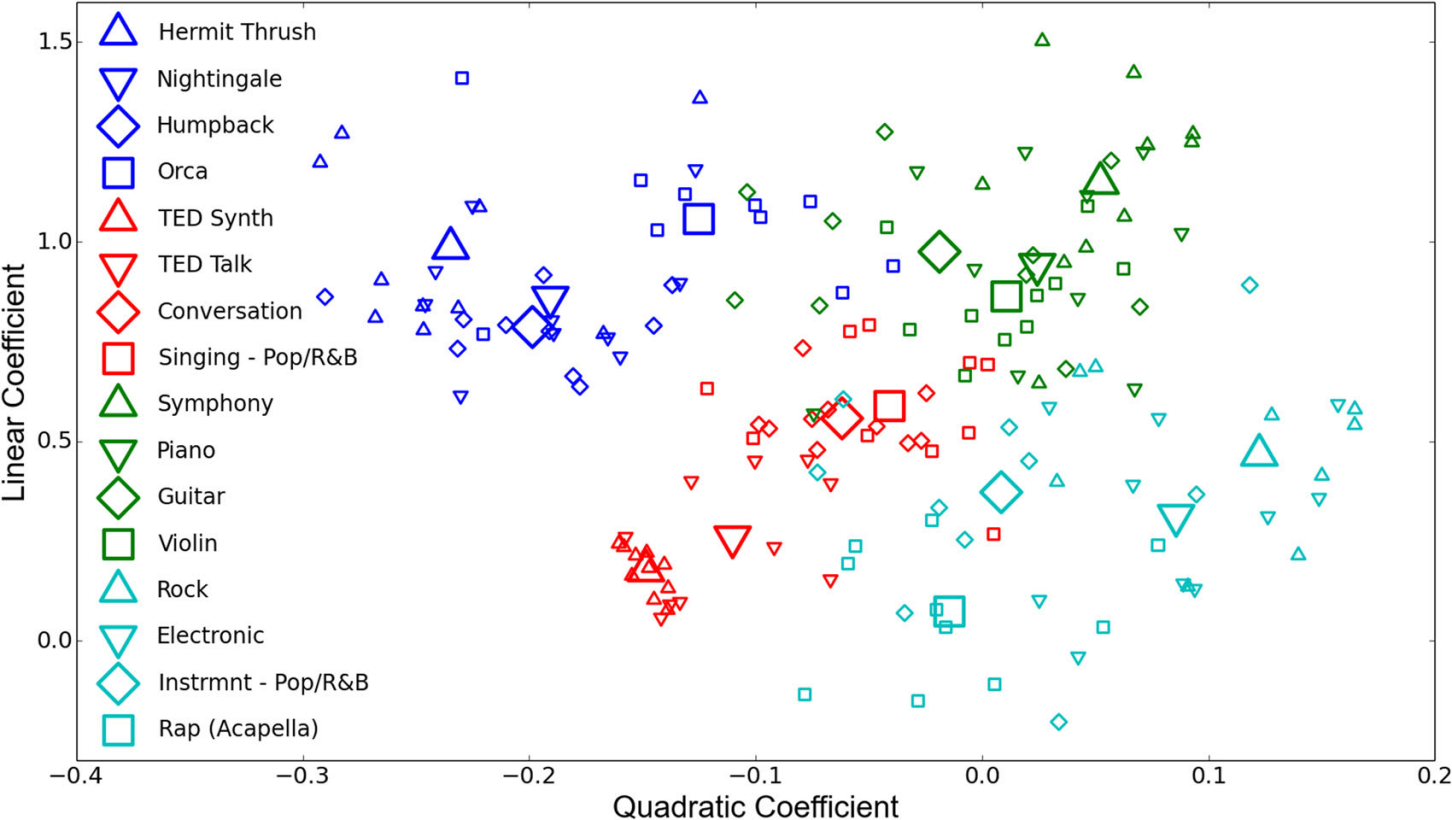
Each AF function for each sound recording from Ref. 152 was quantified in terms of a linear coefficient that corresponded to the degree of hierarchical temporal structure, and a quadratic coefficient that corresponded to the amount and direction of change in hierarchical temporal structure as a function of timescale. Four different categories of sound recordings were analyzed—animal vocalization, speech, classical music, and popular music—each with four subcategories (see the legend). The scatter plot shows a point representing the curvature (*x*‐axis) and slope (*y*‐axis) of the AF function for each of 10‐example recordings per subcategory. One can see that the four main categories have mostly distinct hierarchical temporal structures as measured by AF functions, and in some cases, the subcategories are further distinguished within the main categories. Large symbols indicate the centroid of each subcategory. Figure reproduced verbatim from Ref. 152.

Kello and colleagues^152^ also applied AF analysis to a wide range of speech, music, and animal vocalization recordings. Results were consistent with those of Falk and Kello^134^ and also extended them by showing that nested clustering is enhanced by musical composition. Moreover, AF functions were classified using support vector machines, and the results revealed a natural taxonomy of complex acoustic signals, where recordings within a given category yielded AF functions that could be separated from other categories (Fig. 4).

The AF function category most relevant to the current discussion corresponds to *conversational interactions*. In Ref. 152 and two subsequent studies (Ref. 153 and Schneider, Ramirez‐Aristizabal, Gavilan, and Kello, unpublished data) dozens of recordings of various types of conversational interactions, in both English and Spanish, have all yielded AF functions with a common slope and bend. The same slope and bend was found for jazz improvisations, which have been likened to conversations.^154^ Most notably, recordings of animal vocalizations from killer whales communicating with each other in pods yielded AF functions with the same basic shape as those for recordings of conversational interactions. Animal vocalizations from humpback whales, nightingales, and hermit thrushes were different—these animals do not use their songs in the service of vocal interactions, and AF functions did not follow the pattern common to conversational interactions. Instead, these other animal vocalizations fell into their own distinct pattern, closer to a monologue or solo song in terms of hierarchical temporal structure. Ravignani and colleagues^44^ applied the same AF analysis to recordings of harbor seal pups, a species that employs vocal interactions similar to killer whales, and these recordings also yielded the same communicative AF function shape.

The observed commonality in so many different recordings of communicative interactions suggests an intriguing hypothesis: both human and nonhuman communicative interactions of all kinds may manifest the same, unique kind of hierarchical temporal structure that depends on the particular communicative function, and less so on the species or means of sound production. Such a result, if corroborated, would indicate that speech, music, and animal vocalizations all follow a common pattern of hierarchical temporal structure. If true, this could have implications for both segmentation and learning of incoming communicative stimuli.

### Rhythms as distributions of inter‐event intervals

Wildlife recordings often have contributions from diverse sounds thereby obscuring the signal of interest. Having a low signal to noise ratio limits the applicability of techniques acting directly over the waveform. In these situations, an alternative is to annotate the recordings with the onset or offset times and investigate the temporal structure of these events.^44^, ^155^, ^156^ In this section, we present another method (in addition to the AF analyses above and other techniques described in detail elsewhere^7^) for characterizing and comparing temporal patterns in a series of events that was recently proposed in Ref. 157. This technique consists of characterizing timing as distributions of the logarithm of the inter‐event intervals (IEIs) and comparing the distributions with the symmetric Kullback–Leibler divergence (sKL‐divergence). We explore the scope of this method based on its strength to describe temporal structures in four datasets: random, isochronous, hierarchical, and speech. We start by describing the datasets and then discuss this technique.

The datasets consist of time series of events represented by pulses. The isochronous series has a pulse every 0.2 seconds. The random series is a Poisson process with a rate λ = 12 pulses per second. The hierarchical series is composed of triplets of double pulses. All these artificial sets—random, isochronous, and hierarchical—are 10‐s long with a sampling rate of 1 kHz (i.e., a temporal resolution of 1 millisecond). Additionally, the isochronous and the hierarchical series are jittered with Gaussian noise with a standard deviation of 0.005 seconds. The speech dataset comes from “The north wind and sun dataset” corpus, consisting of recordings of the fable in 18 different languages. For our analysis, we use the position of the syllable centers annotated by Jadoul and colleagues.^158^ This annotated speech dataset contains the syllable centers of all 18 languages.

**Figure 5:**
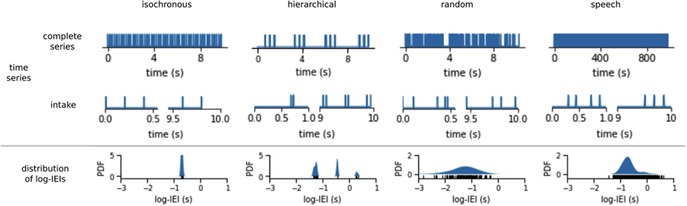
Characterization of the temporal structure of four time series (columns)—isochronous, hierarchical, random, and speech—with distributions of inter‐event intervals (IEIs). The top two rows show the full time series and a “zoom” on (i.e., intake of) the beginning and end of the time series. The bottom row shows the distribution of the logarithm base 10 of the IEI (log‐IEI).

The distribution of the logarithm of the IEI (log‐IEI) highlights the typical IEI in the time series (Fig. 5). The isochronous signal has an event every 0.2 s, so its distribution of log‐IEI shows a single peak at 0.2 s (Fig. 5). The distribution of the random series ranges over various time scales around 1/λ. The hierarchical series presents three peaks—the shortest corresponds to the IEI within the double pulses, the middle one corresponds to the IEI between the double pulses within the triplet, and the third one corresponds to the intervals between the triplets. The IEIs of the speech dataset are spread with 50% of the IEIs between 0.19 and 0.25 seconds.

**Figure 6:**
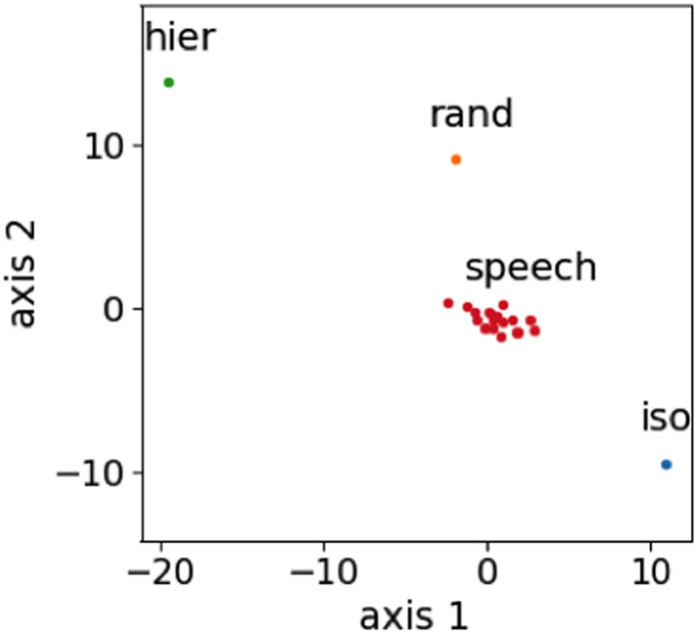
Two‐dimensional scaling of the distances between the distributions of inter‐event intervals (IEI). Pairwise distances computed using the symmetric Kullback–Leibler divergence (sKL‐divergence) and scaled to a two‐dimensional space using Scikit‐learn MDS method.^159^ This method takes pairwise distances between elements and locates them in an *n*‐dimensional space. Here, we have chosen *n* = 2. There is one point for each distribution coming from the datasets isochronous (iso), hierarchical (hier), random (rand), and the 18 speech datasets. Neither the location of the points nor the axes are relevant per se; what counts here are the distances between the points—these are such that they try to preserve the sKL‐divergences.

The downside of the distributions of IEI is that they are not sensitive to high‐order temporal structures, as randomizing the IEIs would yield the same distributions. The advantage of these distributions is that they are easy to interpret and can be compared using the sKL‐divergence (Fig. 6).^157^ The Kullback–Leibler divergence measures the similarity between two probability distributions. The divergence is smaller the more similar two probability distributions are, being zero only for identical distributions.

Comparing the distributions of our datasets using the sKL‐divergence, we obtain 210 distances. By projecting these distances to a two‐dimensional space, we observe that the distributions of the speech datasets are more similar to each other than each is to one of the other datasets (Fig. 6). This approach is described in detail in Ref. 157.

The IEI‐related distributions can be visualized and used for computations by employing either the IEI itself, or its logarithm (as we do here). Using the logarithm is advantageous because it scales the IEI according to their magnitude, thereby dealing with different time scales simultaneously. This logarithmic scaling may also be quite plausible neurobiologically, at least for single intervals and musical rhythm.^3^, ^160^ However, sometimes, one may prefer to work with the IEI directly, for instance, for dealing with negative intervals arising from overlapping calls from different signalers.^161^, ^162^ The fact that this method can work with both IEIs and their logarithm makes it flexible to work with different types of datasets.

## Time and rhythm: linking neural systems and behavior

### From cross‐species comparisons to evolutionary inference

Rhythms comprise features, such as intensity and duration, that fluctuate at somewhat equal time intervals in a complex and continuous auditory signal, such as human speech and music. Yet, an unresolved topic in time and rhythm research is why and how the ability to process temporal and rhythmic structure emerged in humans.^118^, ^119^, ^163^ One idea ties rhythm processing to social synchronization across a number of species (for a review, see Ref. 13). Other research exploring the neurocognitive function of time and rhythm processing also points toward similarities of rhythmic and structural properties in speech and music^164^, ^165^ that are primarily denoted in vocal learners.^108^ This coevolution of properties might reside in, and still rely on, frontostriatal brain circuitry^94^, ^166^ (see also, Ref. 167 on the evolution of structure), a system that engages in and monitors the acquisition of hierarchical pattern formation in multiple domains. This brain system also tags specific longer scale temporal attributes and synchronizes to temporal and structural cues found in speech and music (e.g., Refs. 5 and 168). However, it remains a mystery (1) how humans derived more complex structures in speech, language, and music from the temporal and sequencing properties of the frontostriatal system and (2) where the structural and functional boundaries lie within this system that separate human and nonhuman species. Consequently, a comparative approach to evaluate the computational proximity and extent of temporal and rhythmic sequences in species relying on an extended frontostriatal circuitry is called for.^13^

### Human neurocognitive architecture of time and rhythm processing

The spatiotemporal properties of auditory signals reach the thalamus and cerebellum in the earliest stages of auditory processing. While precise and continuous spatiotemporal information is sent via the thalamus to the auditory cortices where sensory and memory processes are initiated, the cerebellum projects salient events encoded in the auditory signal (onsets, offsets, and sharp energy changes) via the thalamus directly to frontal cortices (e.g., presupplementary motor area (SMA)). This latter trajectory is relevant for two reasons: (1) it attracts and maintains attention to salient changes in the auditory signal and (2) based on this dynamic attention modulation, prepares the frontostriatal system for the encoding of temporal interevent relations (intervals) that form the basic segmentation unit of sequences. The encoding and evaluation of the temporal cohesion of sequences require working memory and rely on the prefrontal cortex (PFC),^169^ where temporal and memory information integrates.^5^

In production, the generation of a sequence engages the PFC. To start and continue this process, an interface of the pre‐SMA and frontostriatal circuitry acts as a “pacemaker” and stabilizes a temporal grid for auditory sequence processing. Sequences adhere to a temporal architecture that integrates fast, short‐range transitioning temporal events via the cerebellum and slower large‐range intervals via the striatum (see also, Ref. 170 for different terminology). The actual initiation, timing, and triggering of auditory−motor sequences as for example found in speech engage the SMA‐proper that controls these processes (e.g., see Refs. 171 and 172), followed by the premotor and primary motor cortices for the execution of sequences.

**Figure 7:**
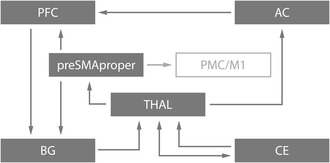
Cortico–subcortico–cortical neural circuitry underlying time and rhythm perception and production. CE, cerebellum; THAL, thalamus; AC, auditory cortex; PFC, prefrontal cortex; BG, basal ganglia; PMC, premotor cortex; M1, primary motor cortex; pre‐SMA, presupplementary motor area. See also, Ref. 177.

In sum, the described temporal architecture (Fig. 7) composed of fast, short‐range and slower, long‐range temporal information contributes both to perception and production of auditory−motor sequences, such as found in human speech and music.^5^, ^173^, ^174^ Empirical evidence confirms that the ascribed temporal properties form the basis of temporal pattern formation found in simple and complex rhythm processing, which also relies on the same neural frontostriatal architecture as temporal processing per se.^175^, ^176^

### Shared neural circuitry, but where are cross‐species boundaries?

While there is now ample evidence that several species of birds and mammals, including some nonhuman primates, rely on comparable frontostriatal circuitry (e.g., see Ref. 178) to acquire and produce simple and slightly more complex temporally structured sequences, vocal learning alone does not suffice to acquire hierarchical temporal structures found in human speech and music.^179^ For example, zebra finches produce temporally structured syllable sequences^22^ and can perceptually group auditory input.^16^ Rhesus monkeys can produce single intervals and synchronize to a metronome,^180^ while macaques display auditory grouping.^181^, ^182^ So far, though there is no evidence that any one of these species can form hierarchical temporal structure as found in human speech and music. One explanation, while still speculative, could be that the strict serial order of events in time does not yet define rule‐based behavior beyond local dependencies.^94^ Second, complex temporal and rule structure building may rely on an intricate relationship between frontostriatal and frontocerebellar circuitry, where the expansion of the neocerebellum reciprocally pushed the evolution of neocortex, such as the PFC.^183^, ^184^ This latter structural development is considered crucial for hierarchical structure building. Consequently, investigation of this frontostriatocerebellar interface in species producing and perceiving basic temporal structure is required to understand similarities and differences between simple and hierarchical temporal structure building in humans and other species.

## General discussion and conclusions

### Connecting fields, disciplines, and methods

This paper is a first attempt to summarize multiple approaches to understand the comparative and evolutionary nature of human speech rhythm. We reviewed how animals from different taxonomic groups can produce and perceive temporal and rhythmic patterns with features relevant to human rhythm. We examined parallels between human and animal infant vocal production and interactive rhythms in order to better understand contributions of rhythm to human speech development. We found that social interaction in several species, including humans, produces a common pattern of temporal structure in vocalizations. We compared several techniques to measure temporal and rhythmic structure, both in human speech and animal vocalizations. We concluded by discussing the neural circuitry underlying speech rhythm and their relationship with nonvocal motor actions.

Admittedly, however, there is a big disconnect among, at least, five areas of scientific knowledge and research: (1) what we know of human speech rhythm, especially from a developmental perspective, (2) knowledge on how animals produce and perceive sounds which can be related to human speech rhythm, (3) techniques we can use to measure vocal rhythms behaviorally, within humans and across species, (4) comparative work on rhythmic, nonvocal movement, and (5) how our knowledge of the human nervous system relates to that of other species with respect to speech rhythm.

### Future work

We suggest that future work should keep the current sparseness of these five approaches in mind and actively build bridges across them. Pragmatically, this would translate into designing experiments which span two, or more, of the five still loosely connected areas discussed above. For instance, researchers in animal bioacoustics (area 2 above) could perform analyses that are tightly matched to human vocal development (area 1). Likewise, behavioral metrics of vocal rhythmicity (area 4) would be even more valuable if usable as potential markers of neural processes or pathologies (area 5).

A recent, successful example of this kind of multidisciplinary work focused on rhythmic interactivity in a rodent.^26^ Temporal features of songs of Alston's singing mice were investigated. The question was whether these features varied between isolated and interactive singing and were partly controlled by the cortex (as opposed to fully originating from subcortical structures of the brain). The authors of the study provided behavioral, pharmacological, and neural evidence that rhythmic vocal interactivity in Alston's mice stems from a cortico−subcortical circuitry. How this murine circuitry relates to the human circuitry in Figure 7 is still unknown. In addition to introducing this broad methodological approach,^26^ the research tackled comparatively the question of how cortical control of vocalizations and turn‐taking evolved in humans. Therefore, apart from its scientific contribution, this study shows that combining two or more of the areas above is indeed possible.

Two additional areas for future research, not discussed here, include the biology−culture interplay in, and the genetics of, speech rhythm. Studying the biology−culture interface can be used to reconcile old, unproductive nature versus nurture debates by potentially showing how cognitive biases and cultural transmission interact to deliver the rhythmic structure of speech. One possible method to study the biology−culture interplay in the laboratory is the iterated learning paradigm, where each participant learns a behavior which was produced (and modified) by a previous participant who learnt it the same way. Iterated learning experiments have been done to better understand linguistic morphology,^185^ poetry,^186^ and musical rhythm.^160^, ^187^^−^ 189 Iterated learning experiments where participants imitate and transmit nonsense syllable sequences^190^ could be used to show whether, and if so how, cultural transmission amplifies domain‐general biases resulting in rhythmic patterns of speech. This iterated learning approach can be integrated with neurophysiological measures.^191^ Complementarily, tools and methodologies from genetics can be used to map the population genotypes to behavioral variability in rhythmic traits.^19^, ^192^ Initial work has been undertaken in special populations (e.g., those affected by Williams syndrome^193^, ^194^), but could be extended to the whole population of one species, humans, or otherwise.

To conclude, the field of comparative rhythm research is rapidly growing and needs a multidisciplinary approach. This research field offers many low‐hanging fruits, which are ready to be seized by colleagues interested in joining us.

## Competing interests

The authors declare no competing interests.
